# Focus on PTEN Regulation

**DOI:** 10.3389/fonc.2015.00166

**Published:** 2015-07-27

**Authors:** Miriam Bermúdez Brito, Evangelia Goulielmaki, Evangelia A. Papakonstanti

**Affiliations:** ^1^Department of Biochemistry, School of Medicine, University of Crete, Heraklion, Greece

**Keywords:** PTEN, transcription, translation, protein interactions, localization

## Abstract

The role of phosphatase and tensin homolog on chromosome 10 (PTEN) as a tumor suppressor has been for a long time attributed to its lipid phosphatase activity against PI(3,4,5)P_3_, the phospholipid product of the class I PI3Ks. Besides its traditional role as a lipid phosphatase at the plasma membrane, a wealth of data has shown that PTEN can function independently of its phosphatase activity and that PTEN also exists and plays a role in the nucleus, in cytoplasmic organelles, and extracellularly. Accumulating evidence has shed light on diverse physiological functions of PTEN, which are accompanied by a complex regulation of its expression and activity. PTEN levels and function are regulated transcriptionally, post-transcriptionally, and post-translationally. PTEN is also sensitive to regulation by its interacting proteins and its localization. Herein, we summarize the current knowledge on mechanisms that regulate the expression and enzymatic activity of PTEN and its role in human diseases.

## Introduction

Phosphatase and tensin homolog on chromosome 10 (*PTEN*) is a potent tumor suppressor gene located at chromosome 10q23.31 (1) that was discovered in 1997 (2–4). *PTEN* encodes a phosphatase with dual activity against phospholipids and proteins (5). PTEN protein can also regulate signal transduction pathways by both phosphatase-dependent and -independent mechanisms (6). Despite its potential tyrosine and serine/threonine phosphatase activity, the tumor-suppressor activity of PTEN is attributed to its lipid phosphatase activity (7).

Phosphatase and tensin homolog on chromosome 10 is considered the main negative regulator of class I phosphoinositide-3-kinases (PI3Ks) (8). Class IA PI3Ks are heterodimers comprised a 110-kDa catalytic subunit (p110α, p110β, and p110δ) and a regulatory p85 (p85α, p55α, p50α, p85β, or p55γ, collectively called “p85s”) subunit (9, 10) which mediates binding to receptors, activation, and localization of the enzyme (11, 12). p110γ is the class IB PI3K, which occurs in complex with the p101 (13, 14) or p84 (15, 16) protein. PI3Ks are stimulated by a variety of signals, including growth factors and G-protein-coupled receptors (17). When activated, PI3Ks phosphorylate the 3-position of the inositol ring of the phosphatidylinositol-4,5-biphosphate (PIP_2_) to produce the second messenger phosphatidylinositol-3,4,5-triphosphate (PIP_3_) (18). The PI3K complex, which is recruited to the plasma membrane, then activates the pyruvate dehydrogenase kinase 1 (PDK1) and Akt (19). PTEN, through its lipid phosphatase activity, catalyzes the conversion of PIP_3_ to PIP_2_ by dephosphorylating the 3-position of the inositol ring of PIP_3_, antagonizing thus the PI3K signaling (20, 21). Consequently, PTEN blocks the activation of downstream signaling events, including PDK1/Akt and Akt/mammalian target of rapamycin (mTOR) (20). Given that PI3K regulates diverse cellular processes, including cell proliferation and survival, reduced or lost activity of PTEN contributes to the constitutive activation of the PI3K pathway (22–25). The elevated levels of PIP_3_ in cancer cells prevent apoptosis and moreover allow an uncontrolled cellular growth and proliferation (26). Many human cancers are associated with somatic deletions or mutations of the PTEN gene, including endometrial carcinoma (27, 28), glioblastoma multiforme (29, 30), skin (31), breast (32, 33), and prostate cancers (34). Moreover, PTEN is being emerging as an important factor in other diseases, such as diabetes (35) and autism spectrum disorders (36).

Phosphatase and tensin homolog on chromosome 10 plays a key role as tumor suppressor, therefore maintaining a stable level of its expression seems to be critical. Studies have shown that the expression and enzymatic activity of PTEN are tightly regulated at transcriptional, post-transcriptional, and post-translational level, as well as by protein–protein interactions (37, 38). The understanding of how PTEN is regulated is essential for deepening our knowledge into cancer biology that could provide new strategies for cancer therapies. In this review, we go over the main insights into PTEN regulation.

## Transcriptional Regulation

**Figure 1:**
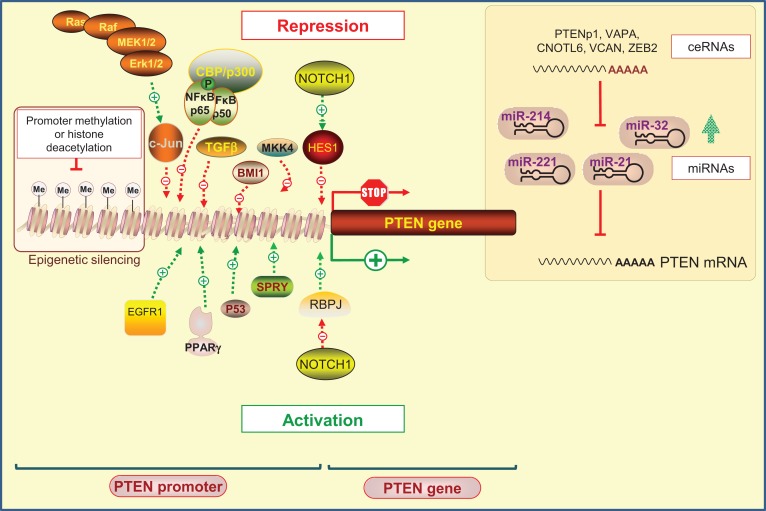
**Regulation of PTEN expression by epigenetic, transcriptional, and post-transcriptional mechanisms**. Positive regulators of PTEN gene expression include early growth response protein-1 (EGFR-1), peroxisome proliferator-activated receptor-γ (PPAR-γ), tumor protein 53 (Tp53; also known as p53), and human sprout homolog 2 (SPRY2). PTEN transcription can also be repressed. Several transcription factors including c-Jun, nuclear factor kappa B (NF-κB), mitogen-activated protein kinase kinase-4 (MKK4), transforming growth factor beta (TGF-β), and the polycomb group protein BMI1 are known to repress PTEN expression. Interestingly, it has been shown that NOTCH signaling could be either a negative or positive regulator of PTEN transcription targeting the transcription factor hairy and enhancer of split1 (HES1) or the recombining binding protein suppressor of hairless (RBPJ, also known as CBF-1), respectively. Epigenetic silencing by the gene promoter methylation and histone modifications may also negatively control PTEN expression. At the post-transcriptional level, a variety of miRNAs have been identified to repress PTEN mRNA translation. An additional level exists, involving endogenous non-coding transcripts that have regulatory functions by quenching miRNAs, such as PTENpg1 and ZEB2.

Phosphatase and tensin homolog on chromosome 10 promoter is positively and negatively regulated by many transcription factors that operate at specific times and in different types of cells (37) (Figure 1).

Positive regulators of *PTEN* gene expression include early growth response protein 1 (EGFR-1), peroxisome proliferator-activated receptor γ (PPAR-γ), and tumor protein 53 (Tp53; also known as p53). These transcription factors were shown to directly bind to PTEN promoter region (39–41). The human sprout homolog 2 (SPRY2) mediates also its anti-proliferative actions by altering PTEN content and activity (42). On the other hand, diverse negative regulators of PTEN have been reported. Indeed, the Ras/Raf/MEK/ERK pathway was shown to suppress the transcription of PTEN through c-Jun (43). Likewise, nuclear factor kappa B (NF-κB) negatively regulates PTEN expression through sequestration of the transcriptional co-activator CBP/p300 (44). In addition, transcription factors, such as mitogen-activated protein kinase kinase-4 (MKK4), transforming growth factor beta (TGF-β), and the polycomb group (PcG) protein BMI1, were reported to suppress PTEN expression in several cancer models (45–48).

Epigenetic silencing by gene promoter methylation and histone modifications has also been proposed as a mechanism by which PTEN expression can be suppressed. This mechanism of PTEN regulation has been implicated in several cancers, such as gastric, colorectal, melanoma, and breast cancer (49–52). Several studies have shown that silencing of PTEN transcription is often due to the presence of hypermethylated CpG islands in the PTEN promoter (49, 52). However, careful consideration must be taken when interpreting epigenetic silencing regarding PTEN because a PTEN-pseudogene exists with a promoter, which is also shown to be methylated (53). PTENP1 pseudogene shares 97.8% sequence identity with the PTEN mRNA sequence, and 91% identity with a 921 bp region of the *PTEN* CpG island. Moreover, PTEN shares a CpG island promoter with another gene known as *KLLN*, which is transcribed from the negative DNA strand in the opposite direction (54). Thus, methods that detect methylation but do not distinguish between *PTEN* and the pseudogene sequences may lead to false positives (53). In this scenario, the methylation status of regions with particularly high homology to pseudogenes can only be determined using allelic bisulfite sequencing to allow the identification of pseudogene-specific alleles (54, 55).

Finally, NOTCH signaling has been shown to act either as a negative or as a positive regulator of PTEN transcription. By acting through PTEN down-regulators, NOTCH signaling leads to PTEN downregulation by activating the transcription factor hairy and enhancer of split1 (HES1) (56), or conversely, to its up-regulation by inhibiting the recombining binding protein suppressor of hairless (RBPJ, also known as CBF-1) (57, 58).

## Post-Transcriptional Regulation

MiRNAs are small and evolutionarily conserved non-coding single-stranded RNAs that negatively regulate gene expression by binding to the 3′-untranslated region (3′-UTR) of the target mRNAs in mammalian cells, leading to mRNA degradation or translational repression (59–61). miRNAs are thought to regulate the expression of almost all genes and consequently to play critical roles in the coordination of fundamental processes, including differentiation, proliferation, angiogenesis, death, and metabolism (59, 62, 63). Increasing evidences have demonstrated that miRNAs play an important role in the pathogenesis, from initiation to metastasis, of many cancers by regulating proto-oncogenes or tumor-suppressor genes (64, 65). Moreover, miRNAs that act as tumor suppressors (e.g., miR-145, miR-124, and miR-142–3p) (66–68) or oncogenes (e.g., miR-21, miR-218, and miR-24) (69–71) have been identified in many types of tumors.

Given the importance of PTEN as a tumor suppressor (72), it is not surprising that a plethora of miRNAs have been identified to modulate PTEN expression at the post-transcriptional level (Figure 1). These miRNAs include those that contain a single harpin structure, such as *miRNA-21* (73, 74), *miRNA-22* (75), and *miRNA-214* (76), as well as those with a polycistronic structure, such as *mir-17–92* (77–79), *mir-106b-25* (75), *mir-367–302b* (75), and *mir-221–222* (74).

miR-21 is one of the most frequently aberrant miRNAs in human cancers. Upregulation of miR-21 has been reported to directly target PTEN promoting thus the growth and metastasis of specific cancers, including non-small cell lung cancer (80), colorectal carcinoma (81), ovarian cancer (82), as well as triple-negative breast cancers (83). Furthermore, Iliopoulos et al. (84) has shown that miR-21, together with miR-181b-1, inhibit PTEN and cylindromatosis (CYLD) tumor suppressor functions, respectively, leading to increased NF-kB activity thus underlying the epigenetic switch that links inflammation to cancer. miR-214 was found to induce cell survival and cisplatin resistance by direct binding to the 3′-UTR of PTEN leading to decreased PTEN expression and to activation of Akt signaling pathway (85). Deregulation of miR-214 has also been shown in several human tumors including breast, melanoma, and hepatocellular cancer (86). In addition, miR-93 and miR-130a may also be associated with cisplatin resistance by direct targeting PTEN in ovarian cancer cells (87, 88). In line with this result, an inhibitor of MiR-130a was found to reverse the cisplatin resistance by upregulating the expression of PTEN and downregulating P-glycoprotein (P-gp) in A2780 cell lines (89).

Comparable findings have also been published for other miRNAs. Recently, Xie et al. (90) reported that miR-221 targets PI3K/Akt signaling axis inducing thus cell proliferation and 1,3-bis(2-chloroethyl)-1-nitrosourea) (BCNU) resistance in human glioblastoma. More specifically, the overexpression of PTEN lacking 3′-UTR or the PI3K inhibitor wortmannin was found to attenuate the miR-221-mediated BCNU resistance and to promote cell apoptosis.

The importance of PTEN regulation by miRNAs in cancer progression is highlighted in multiple occasions. Indeed, deregulation of miR-214, miP-199a*, miR-200a, and miR-100 was shown to be frequent in ovarian cancers (85). MiR-26a negatively regulates the expression of PTEN in a murine glioma model and enhances the formation of tumors *de novo* (91). MiR-26a also enhances lung cancer metastasis via modulation of metastasis-related genes and PTEN inhibition (92). Similarly, Lang et al. (93) showed that miR-429 induces tumorigenesis of human non-small cell lung cancer cells by directly targeting the 3′-UTR of multiple tumor suppressor genes, including PTEN, RASSF8, and TIMP2. In breast cancer, PTEN is targeted by miR-29b (94) and miR-301 (95). miR-301a was also shown to directly target and suppress PTEN, maintaining constitutively activated Wnt/β-catenin signaling, which leads to the enhancement of breast cancer invasion and metastasis (96).

High-frequency miRNA dysfunction is also associated with prostate cancer development and progression. MiR-153 promotes proliferation of human prostate cancer cells through direct suppression of PTEN expression (97). Additionally, a combination of four miRNAs (miR-19b, miR-23b, miR-26a, and miR-92a) was found to regulate PTEN expression post-transcriptionally and to affect the downstream PI3K/Akt pathway via PIK3CA (p110α), PIK3CD (p110δ), PIK3R1 (p85), Akt, and cyclin D1, thus promoting prostate cancer cells proliferation *in vitro* (98).

The regulation of PTEN by miRNAs is also found in colorectal cancer (99). miR-92a was reported to promote cell metastasis of colorectal cancer through the PTEN-mediated PI3K/Akt pathway. Likewise, upregulated miR-494 was found to directly target the 3′-UTR of PTEN and this was associated with tumor aggressiveness and tumor metastasis (100). miR-103 was shown to promote colorectal cancer through downregulation of the tumor-suppressor genes DICER and PTEN (101). On the other hand, miR-32 is overexpressed in colorectal carcinoma inducing cell proliferation, migration, and invasion (102). Moreover, the expression of miR-32 was upregulated in hepatocellular carcinoma tissues and cell lines and inversely the expression of PTEN was decreased (103).

Emerging evidence has highlighted the importance of new types of RNA–RNA interactions that underlie the regulation of gene expression. It is important to note that one single miRNA can regulate multiple mRNAs of the same or different pathways and thereby a single miRNA can control an entire post-transcriptional program and influence dozens of target genes (104). Conversely, several miRNAs can regulate a single mRNA. Moreover, RNA transcripts, such as mRNAs, non-coding RNAs, pseudogene transcripts, and circular RNAs, could regulate each other by competing for the same pool of miRNAs, acting as “competing endogenous RNAs” (ceRNAs) (105, 106). These ceRNAs share sequences recognized by the miRNAs called microRNA recognition elements (MREs) (107). ceRNAs could sponge the miRNA through direct competition for miRNA binding and as a consequence increase the levels of endogenous miRNA targets (108). More recently, ceRNAs that take crucial roles in oncogenic pathways of many types of cancer have been called “oncocers” (109).

The first evidence that an endogenous non-coding transcript can have regulatory functions by quenching miRNAs in humans was presented by Poliseno et al. (110). PTEN expression was found to be post-transcriptionally regulated by the action of a *PTEN* pseudogene (*PTENpg1*, also known as *PTENp1*, *PTEN2*, and *PTEN*Ψ*1*) network (110). The *PTENp1* locus encodes three different long non-coding RNA (lncRNA) molecules, one sense *PTENp1* and two functional antisense RNAs (asRNAs) isoforms, α and β (111). *PTENp1* sense is an lncRNA that shares extensive sequence homology with PTEN mRNA, especially in the ORF region and within the first third of its 3′-UTR, a region enriched for known miRNA target sites. Thus, it functions like an miRNA sponge, sequestering them and therefore de-repressing PTEN expression and enhancing its tumor-suppressor activity. For instance, PTENp1 was found to sequester several miRNAs families that target PTEN mRNA, such as miR-17, miR-19, miR-21, miR-26, and miR-214, among others (110). An additional level of complexity in this crosstalk between miRNAs and RNAs is provided by PTENp1 antisense transcripts, which regulate PTEN expression both at the transcriptional and translational levels. *PTENp1* as RNA-α acts in *trans*, localizes to the PTEN promoter, and inhibits PTEN transcription by recruiting epigenetic repressor complexes. In contrast, the β isoform, which is partially complementary to *PTENp1* sense, interacts with *PTENpg1* sense through an RNA:RNA pairing interaction, which positively regulates PTEN expression, and thus promotes stabilization of *PTENp1* sense by binding to its 5′ end (111). PTENp1 is not the only miRNA decoy regulating PTEN expression. Other protein-coding genes, such as vesicle-associated membrane protein-associated protein A (VAPA), CCR4-NOT transcription complex, subunit 6-like (CNOT6L), or Versican (VCAN), have a similar role and act as competing RNAs for PTEN-interacting miRNAs (112, 113). Moreover, Karreth et al. (114) characterized ZEB2 as a *bona fide* PTEN ceRNA. ZEB2 acts a tumor suppressor by regulating PTEN expression through its mRNA in melanoma. The abrogation of ZEB2 expression releases the miRNAs that downregulate PTEN, cooperating with BRAF to promote melanomagenesis.

New miRNAs that are involved in PTEN regulation are still being reported and the functions of most of these miRNAs still remain to be discovered. Direct targeting of miRNAs might be a potential strategy of certain cancer treatments in the near future.

## Post-Translational Modifications of PTEN

**Figure 2:**
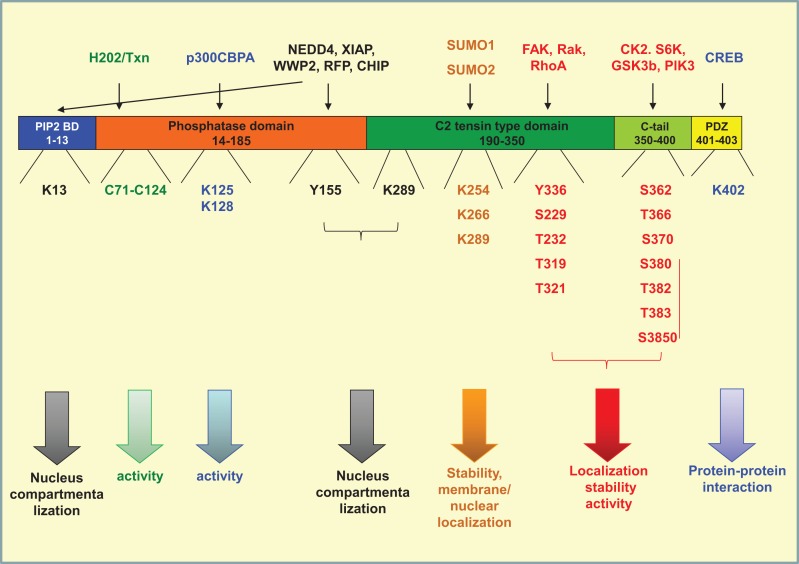
**Post-translational modifications of PTEN**. PTEN is composed of four structural domains [PIP2-binding domain (aa 1–13), a catalytic tensin-type phosphatase domain (aa 14–185), a C2 tensin-type domain, which binds phospholipids (aa 190–350), the carboxy-terminal tail of the protein (aa 350–400), and a PDZ-binding domain (aa 401–403). The residues where phosphorylation (red), acetylation (blue), oxidation (green), ubiquitination (black), and sumoylation (orange) take place are indicated. The proteins conferring each modification are mentioned on the top of the figure. All modifications interfere directly or indirectly with the activity of the phosphatase by regulating also its compartmentalization and/or its stability.

Phosphatase and tensin homolog on chromosome 10 is a 403-amino acid protein composed of four structural–functional domains (115). A PI(4,5)P_2_ (PIP2) binding domain (aa 1–13), a catalytic tensin-type phosphatase domain (aa 14–185), a C2 tensin-type domain, which binds phospholipids (aa 190–350), the carboxy-terminal tail of the protein (aa 350–400), and a PDZ-domain binding (aa 401–403). Modifications of amino acids in each one of the above domains interfere with protein stability, activity, interaction with other proteins, and localization (Figure 2).

### Phosphorylation

Phosphatase and tensin homolog on chromosome 10 is one of the most commonly mutated tumor suppressors in human cancers. However, loss of PTEN activity in the absence of mutations appears to occur in an even greater number of tumors. This could readily be due to post-translational modifications of the phosphatase. For example, in human T cell acute lymphoblastic leukemia (T-ALL), increased phosphorylation of PTEN results in hyperactivation of the PI3/Akt pathway (116, 117). Increased levels of phosphorylated PTEN were also detected in samples from patients with gastric cancer suggesting a role in gastric carcinogenesis (118).

#### C-Tail Phosphorylation

The most well-studied phosphorylation events taking place in PTEN are that in the C-tail moiety of the molecule and more specifically on amino acids Ser362, Thr366, Ser370 (119, 120), and the cluster containing Ser380, Thr382, Thr383, and Ser385 (121, 122). The phosphorylation of the cluster is driven mainly by casein kinase 2 (CK2), as well as by S6K (120, 123), whereas the phosphorylation of Ser362 and Thr366 is conferred by glycogen synthase kinase 3β (GSK3b) (119, 124). Thr366 and Ser370 are phosphorylated by polo-like kinase 3 (PlK3) (125) (Figure 2).

The effect of these phosphorylation events on PTEN protein function is “pluripotent.” Phosphorylation of PTEN interferes with the localization, the stability, and the activity of the protein. C-tail phosphorylated PTEN seems to adopt a compact conformation, which does not allow the protein to interact with membrane phospholipids (126–130) or with other membrane anchored proteins, mainly PDZ-containing proteins, such as MAGI-3 (131–133). Abolishment of PTEN membrane interactions renders the phosphatase inactive against its membrane localized substrate PI(3,4,5)P_3_. The importance of PTEN membrane anchoring has also been shown by mutations affecting the PIP2 lipid-binding domain, which also totally abrogated the PTEN function (127, 134). Recent data reveal an alternative mechanism through which PTEN phosphorylation affects dimerization and activity of the protein. More specifically, two PTEN species were identified; a dimeric unphosphorylated and a monomeric phosphorylated. The monomeric phosphorylated conformation of PTEN was shown to be essentially inactive (135). C-tail phosphorylation of PTEN was shown to affect not only the cytosol/membrane but also the cytosol/nuclear protein distribution. More specifically, phosphorylation of PTEN led to nuclear export of the protein (123, 136).

Interestingly, although PTEN phosphorylation negatively affects the phosphatase activity, it seems to increase the protein stability. More specifically, closed phosphorylated PTEN is less prone to ubiquitin ligases and therefore less prone to degradation in comparison to open unphosphorylated PTEN (122, 133).

It is of note that the phosphorylation events and their effect on protein stability and/or activity may differ according to the cell context. For instance, phosphorylation of Thr366 led to destabilization of the phosphatase in glioma cell lines but not in epithelial cell lines (124).

#### C2 Domain Phosphorylation

Similarly to C-tail, the C2 domain has also been shown to be phosphorylated by different kinases. RAK was shown to phosphorylate Tyr336 whereas RhoA-associated kinase (ROCK) has been shown to phosphorylate Ser229, Thr232, Thr319, and Thr321 (137, 138) (Figure 2). The activity of these kinases seems to affect also PTEN activity, localization, and stability. More specifically, Rak activity prevents the binding of PTEN to its E3 ligase NEDD4-1 thus protecting PTEN from polyubiquitination and subsequent protein degradation (137, 138). On the other hand, ROCK together with the Rho small GTPases, RhoA and Cdc42, regulates PTEN intracellular localization and PTEN-mediated chemotaxis (137, 138). Similarly, PTEN was found to become activated and tyrosine phosphorylated by a RhoA/ROCK-mediated mechanism, which is a part of a p110δ PI3K-induced signaling cascade that leads to the regulation of chemotaxis and cell proliferation (139, 140). We have also recently found that FAK phosphorylates PTEN on Tyr336 (Figure 2) downstream of RhoA and under the negative control of p110δ PI3K (141). The phosphorylation on Tyr336 by FAK affects phosphatase activity, membrane association, and stability of PTEN protein (141).

#### PTEN Dephosphorylation

Dephosphorylation of PTEN is equally important for its biological functions. N-myc downstream-regulated gene 2 (NDRG2) is a PTEN-binding protein that recruits protein phosphatase 2A (PP2A) to PTEN. Expression of NDRG2 is frequently downregulated in adult T-cell leukemia-lymphoma (ATLL), resulting in enhanced phosphorylation in C-tail of PTEN and enhanced activation of the PI3K–AKT pathway (117).

Apart from its lipid phosphatase activity, PTEN also has a poorly characterized protein phosphatase activity. PTEN appears to be able to dephosphorylate itself at its C-tail and C2 domain (142, 143). It could be that the dominant role for protein phosphatase activity of PTEN is autodephosphorylation-mediated regulation of its lipid phosphatase activity.

### Oxidation

Phosphatase and tensin homolog on chromosome 10 belongs to the enzyme family of protein tyrosine phosphatases (PTP). Oxidation of several PTPs by H(2)O(2) forms a disulfide bond between two cysteines, one in the active site (in the case of PTEN Cys 124) and another nearby (Cys71, in the case of PTEN) and followed by consequent inactivation of the enzymes. The disulfide bond likely confers efficiency in the redox regulation of the PTPs (144).

Exogenously and endogenously produced H_2_O_2_ were shown to oxidize and inactivate recombinant PTEN and PTEN produced in cultured macrophages (145). Mice lacking peroxidase Prdx1 produce more ROS and are prone to carcinogenesis. In these mice, Akt pathway is hyperactive and PTEN was found to be inactivated due to increased oxidation (146). Inactivation of PTEN can be reversed by reduction of H(2)O(2)-oxidized phosphatase. This step in cells appears to be mediated predominantly by thioredoxin (147). The latter was also verified by analysis of Thioredoxin-interacting protein (Txnip) knock-out mice. Txnip was shown to be required to maintain sufficient thioredoxin activity in order to reductively reactivate oxidized PTEN and oppose Akt downstream signaling (148). Surprisingly, Thioredoxin-1 was also found to bind directly to the C2 domain of PTEN leading to steric interference of its catalytic site (149).

### Acetylation

Phosphatase and tensin homolog on chromosome 10 function can also be regulated by acetylation and deacetylation events. Two acetylation sites have been identified till now. One is located in the phosphatase active domain (Lys 125–128) and is conferred by p300/calcium-binding PTEN-associated factor. Acetylation of this site was reported to downregulate PTEN activity (150). The other acetylation event is driven by CREB-binding protein and results in the modulation of PTEN interaction with other PDZ-domain containing proteins (151). PTEN was also shown to be deacetylated by histone deacetylase SIRT1 resulting in enhancement of its phosphatase activity (152).

### Ubiquitination

Ubiquitination is an enzyme-catalyzed cascade that is known to mark protein substrates for 26S proteasome-dependent degradation by covalently conjugating them with multiple ubiquitin monomers via lysine (K)48-linkage. Furthermore, ubiquitination also alters protein localization, trafficking, and/or activation via K63-linked polyubiquitin chains. In case of PTEN, both states of mono- and poly-ubiquitinations have been reported. Poly-ubiquitination targets PTEN for proteasomal degradation, which also finely tunes the expression levels of the protein in the cell (122, 133). Mono-ubiquitination takes place at Lys13, Tyr155, and Lys289 and regulates PTEN localization in the nucleus. PTEN compartmentalization in the nucleus is a key regulator of tumor development (153). In parallel, it was shown that both poly- and mono-ubiquitination of PTEN inhibit its phosphatase activity, thus interfering also with the tumor-suppressive role of PTEN (124).

Several enzymes were found to catalyze the ubiquitination of PTEN. NEDD4 was identified as a potential ubiquitin ligase of the phosphatase in some cases (154, 155) but not all (156), although its role in tumor development may be independent of PTEN (157). Other E3 ubiquitin ligases that found to interact with PTEN are XIAP (158), WWP2 (159), and RFP (160) as well as the chaperone-associated E3 ligase and C terminus of Hsc70-interacting protein (CHIP), which also plays the role of an E3 ligase (161) (Figure 2).

Importantly, ubiquitination is a reversible state; deubiquitination is conferred by the PML–DAXX–HAUSP network and is proposed to act as an additional switch controlling PTEN activity, expression levels, and compartmentalization (162). Besides ubiquitination, another frequent post-translational modification occurring at lysine residues is SUMOylation. PTEN is modified by the small ubiquitin-like proteins, small ubiquitin-related modifier 1 (SUMO1), and SUMO2 at lysine residues 254, 266, and 289, all of which are located in the C2 domain (163, 164) (Figure 2) These modifications were shown to enhance PTEN interaction with the membrane and to regulate nuclear localization (165), whereas in parallel SUMOylated PTEN is less prone to ubiquitination (163).

## Regulation of PTEN by Protein Interactions

**Figure 3:**
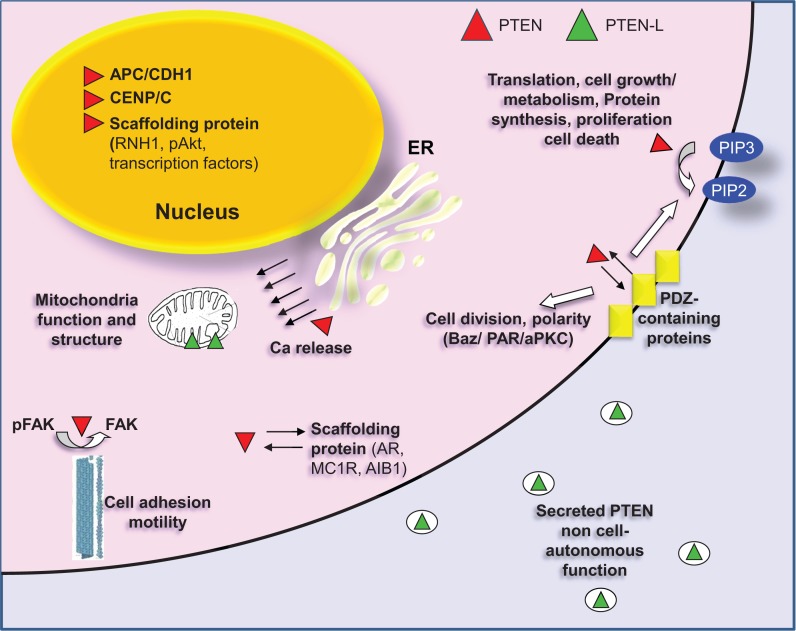
**PTEN localization and interactions with representative partners**. Canonical PTEN (red) can be found in the cytoplasm or in cytoplasmic organelles (ER, mitochondria) and in the nucleus. The primary cellular substrate of PTEN is the second messenger phosphatidylinositol (3,4,5)-triphosphate (PIP3), which hydrolyzes giving phosphatidylinositol (4,5)-biphosphate (PIP2), thus regulating the PI3K pathway. PTEN needs to be recruited on the membrane to perform this function. The association with the membrane is regulated, among others, by interaction with PDZ-domain proteins (MAGI1/2/3). PDZ interaction between PTEN and Bazooka (a PDZ-domain containing protein) is also important for the formation of the PAR/aPKC complex, which is required for the separation of apical and basolateral plasma membrane domains, for the asymmetric localization of cell fate determinants and for the proper orientation of the mitotic spindle. In the cytoplasm, PTEN also dephosphorylates FAK thus inhibiting cell adhesion. Finally, cytoplasmic PTEN also functions as a scaffolding protein. It associates with androgen receptor (AR), with melanocortin-1 receptor (MC1R) as well as with Amplified in breast cancer 1 (AIB1), a transcriptional coactivator. In all cases PTEN regulates the stability of these proteins by promoting their degradation. In the nucleus, PTEN also functions as a scaffolding protein. It interacts with transcription factors regulating their activity [p53, SMAD3, MTF-1 (metal-responsive transcription factor 1)]. It correlates with miRNA processing by interacting with RNH1 and interfering with the formation of Drosha complex and it promotes degradation of nuclear located proteins as in the case of pAKT. Nuclear PTEN interacts also with CENP/C promoting chromosomal integrity and positively regulates expression of Rad51, which reduces the incidence of spontaneous double-strand breaks (DSBs). Additionally, PTEN is required in the nucleus for the formation of the APC/CDH1 complex, which is a regulator of cellular senescence and cell cycle. Canonical PTEN located in the ER is associated with Ca^2+^ release and regulation of apoptosis through interaction with the inositol 1,4,5-trisphosphate receptors (IP3Rs). A translational variant of PTEN (PTEN-L, green) is secreted and associated with the mitochondria. PTEN-L exerts its phosphatase activity outside the cell boundaries, whereas in mitochondria it is related to mitochondria structure and energy production.

The interaction of PTEN with other protein components can lead to a series of post-translational modifications, which heavily modulate the activity of the phosphatase. Reversibly, PTEN also affects the mode of action of its interacting partners. PTEN interactomics is beginning to become delineated by different approaches, either high-throughput proteomics or targeted biochemical assays. The binding partners of PTEN can be divided in different categories. Some representative interacting partners of PTEN are shown in Figure 3.

### Receptors

Sphingosine 1-phosphate (S1PR) (166) and melanocortin-1 (MC1R) (167) receptors, which are G-protein-coupled receptors as well as androgen receptor (AR), which is a nuclear receptor (168), were found to associate with PTEN. In the case of S1PR, PTEN seems to be an intermediate in the signaling pathway regulating cell migration. Apparently, the receptor modulates the phosphorylation of PTEN through the activity of RhoA (166). MC1R is one of the key proteins involved in regulating mammalian skin and hair color. It is mainly expressed in melanocytes and was found to be involved in melanoma development (169). A series of mutations that have been identified in the MC1R gene disable the receptor from binding to PTEN thus permitting PTEN degradation by the WWP2 E3 ligase (167). PTEN also interacts with AR but in this case, the phosphatase prevents the receptor from being translocated to the nucleus and promotes its degradation in the cytoplasm (168).

### Metabolism

Phosphatase and tensin homolog on chromosome 10 interacts with cytosolic α-mannosidase 2C1 (MAN2C1), which is a catabolic enzyme for the breakdown of free oligosaccharides. Interaction of the enzyme with the PTEN seems to inhibit its phosphatase activity in prostate cancer cells (170).

Yeast two hybrid assays also revealed another interesting binding partner for PTEN involved in lipid metabolism, that is fatty acid binding protein 4 (FABP4) (171). FABP4 is mainly expressed in adipocyte tissue. These kinds of interactions point out additional roles for PTEN apart from cancer.

### Cytoskeletal proteins

Phosphatase and tensin homolog on chromosome 10 was found to interact directly with myosin and this interaction seems to be under the control of GSK2/GSK3-kinases. Abolishment of this interaction led to an increase in the neuronal soma size, which is the same phenotype acquired when PTEN function is lost (172).

### Kinases

The interaction of PTEN with CK2, S6K, GSK3, as well as with PlK, RAK, and ROCK is described above.

Given that PTEN catalyzes the dephosphorylation of PI(3,4,5)P_3_, which is required for Akt phosphorylation, it is anticipated that a role of PTEN would be to protect Akt from *de novo* phosphorylation. Surprisingly, PTEN has been shown to function as a scaffolding protein in the nuclei of the cells where it interacts with nuclear phosphorylated Akt leading to its depletion from the nucleus, most likely through proteasomal degradation (173).

An additional interaction of PTEN has been revealed in *Drosophila melanogaster* (174). The Ser/Thr kinase aPKC and the PDZ-domain protein Bazooka form a complex (PAR/aPKC complex), which is required for the separation of apical and basolateral plasma membrane domains, for the asymmetric localization of cell fate determinants and for the proper orientation of the mitotic spindle. PTEN was found to associate and co-localize with Baz and further data revealed that it exerts its function through the actin cytoskeleton (174).

Phosphatase and tensin homolog on chromosome 10 can also directly interact with and reduce adhesion-mediated Tyr-phosphorylation of FAK (175). A key event during cell adhesion is the autophosphorylation of FAK at Tyr397, which is activated upon integrin-clustering. The Tyr397 autophosphorylation site is important for the interaction between FAK and PTEN, and this site has been shown to be dephosphorylated by PTEN. FAK dephosphorylation by PTEN appears to be dependent on cell attachment to specific ECM components (collagenI/IV). The involvement of PTEN in cell adhesion, motility, and invasion could be related to its role as a tumor suppressor gene product in various cell types (176).

LKB1 encodes a serine/threonine kinase generally inactivated in patients with Peutz–Jeghers syndrome (PJS). PTEN was identified as an LKB1-interacting protein. Several LKB1 point mutations associated with PJS disrupt the interaction of LKB1with PTEN suggesting that the loss of this interaction might contribute to PJS. *In vitro* data suggest that PTEN is a substrate of the kinase LKB1, whereas PTEN and LKB1 interaction leads to a cytoplasmic relocalization of LKB1 (177).

### Membrane-associated proteins

Phosphatase and tensin homolog on chromosome 10 usually associates with membrane through PDZ-domain interaction. PDZ domains that bind to PTEN include the PDZ-2 domain from the scaffolding proteins MAGI-1/2/3 (133, 178, 179), the PDZ-2 domain from the human homolog of the *Drosophila* Dlg, hDlg/SAP97 (180), and the unique PDZ domain from the Ser/Thr kinase MAST205 (181). PTEN also binds to the PDZ domains from the MAST205-related protein kinases, SAST and MAST3 (182). PDZ-domain binding is suggested to have a regulatory role on PTEN function by controlling its stability and phosphorylation status.

Co-immunoprecipitation experiments also demonstrated that PTEN among other phosphatases form molecular complexes with caveolin-1 *in vivo* (183). PTEN can be recruited in caveolin rich membrane fractions where it exerts its functional role.

### Transcription factors

Phosphatase and tensin homolog on chromosome 10 is known to play a role in the regulation of numerous transcription factors. Metal-responsive transcription factor 1 (MTF-1) is a mammalian protein, which is essential for embryonic development and it modulates the expression of genes involved in zinc homeostasis and oxidative stress response. PTEN interacts via its phosphatase/C2 domain with MTF-1 and enhances its activity. This regulation is independent of the nuclear translocation, protein stability, or DNA-binding activity of MTF-1 but is promoted by the addition of zinc ions (184).

Phosphatase and tensin homolog on chromosome 10 is the second most mutated tumor-suppressor gene other than p53. Interestingly, PTEN and p53 physically associate. This interaction leads to a significant increase in p53 stability and regulates the transcriptional activity of p53 by modulating its DNA-binding activity (185).

Amplified in breast cancer 1 (AIB1) is a transcriptional coactivator that regulates the transcriptional activities of nuclear receptors and other transcription factors. PTEN interacts with AIB1 and affects its transcriptional activity by enhancing its ubiquitin-mediated degradation. More specifically, PTEN interacts with Fbw7alpha, an E3 ubiquitin ligase, acting as a bridge between AIB1 and Fbw7alpha. This leads to enhanced degradation of AIB1, which eventually accounts for its decreased transcriptional activity (186).

Last but not least, PTEN was found to interact with SMAD3 transcription factor acting downstream of TGF-beta transforming growth factor. TGF-beta promotes tumor progression through induction of tumor invasion, neoangiogenesis, and immunosuppression (187). Published data suggest that PTEN interacts with SMAD3 suppressing its transcriptional activity (188). It could be that loss of PTEN expression in advanced stages of human cancers may contribute to a role for TGF-beta as a tumor enhancer through the SMAD3 signaling cascade (188).

### Nuclear located proteins

The PcG *BMI1* gene maintains the proliferation potential and self-renewal of hematopoietic and neural stem cells and is considered a significant oncogene. PTEN binds to BMI1 exclusively in the nucleus and reduces its function. This interaction does not require the phosphatase activity of PTEN (189).

Phosphatase and tensin homolog on chromosome 10 was also shown to negatively regulate the expression of oncogenic miR-21 at the post-transcriptional level. A microRNA molecule is synthesized as a long RNA primary transcript known as a *pri-miRNA*, which is cleaved by Drosha in the nucleus to produce a characteristic stem-loop structure of about 70 bp long, known as a *pre-miRNA*. RNH1 is an RNA-interacting protein, which was shown to interact with Drosha. This interaction was necessary and sufficient for miR-21 processing and it was shown to be blocked due to PTEN–RNH1 interaction (190).

Besides its well characterized role as a lipid phosphatase, PTEN is considered a guardian of genomic stability. PTEN maintains chromosomal integrity by physical interaction with CENP-C, which is a constitutive protein component of the centromere and is essential for centromere formation. PTEN was also found to be localized in the centromere, probably due to its association with CENP-C, and loss of its expression led to extensive centromere breakage. The PTEN–centromere interaction is independent of its phosphatase activity (191).

Phosphatase and tensin homolog on chromosome 10 was also shown to interact with the anaphase promoting complex/cyclosome (APC/CDH1), which is a ubiquitin E3–ligase complex located in the nucleus and controls the degradation of cell-cycle regulators, such as cyclin, securin, Aurora, etc (192). This interaction is necessary for the formation of the APC/CDH1 complex and it seems to be independent of the phosphatase activity of PTEN (193).

### PTEN regulators

Phosphatase and tensin homolog on chromosome 10 function was shown to be regulated by two proteins; shank-interacting protein-like 1 (SIPL1), which is a member of the NF-κB-activating linear ubiquitin chain assembly complex and protein interacting with carboxyl terminus 1 (PICT-1), which is localized to the nucleus and/or nucleolus. SIPL1 interacts with PTEN through its ubiquitin-like domain (UBL), inhibiting its phosphatase activity (194), whereas PICT-1 binds to the C terminus of PTEN and promotes its phosphorylation and stability (195).

## PTEN Localization

As discussed above, PTEN was shown to localize between the cytosol and the plasma membrane and in the nucleus where it is proposed to play a role in the stability of the genome. More data support that PTEN can also be found extracellularly as well as in cytoplasmic organelles [ER, mitochondria-associated membranes (MAM), and mitochondria] (196) (Figure 3).

### Plasma membrane

Despite the fact that PTEN is required to bind to the membrane to exert its phosphatase activity, it seems that this association is not a steady state situation. Indeed, the N-terminal PIP2-binding motif was shown to drive PTEN on the membrane when PIP2 membrane fraction is enriched (197, 198). Furthermore, the C2 domain of PTEN was found to contain basic residues which are essential for membrane binding (115). However, the association of PTEN to the plasma membrane is regulated by a series of factors. Interaction of the phosphatase with membrane-associated proteins, mainly PDZ-domain proteins, such as MAGI-1, -2, -3 (132, 133, 179), can increase the local PTEN concentration on the membrane (this interaction is discussed above). Apart from that, post-translational modifications also regulate the membrane association of the protein. Such modifications include phosphorylation of the C-tail of PTEN that changes the PTEN conformation and inhibits the membrane binding (126–128, 130, 199) as well as SUMO 1 modification, which was shown to enhance its membrane association (164).

### Nucleus and nucleolus

The function of PTEN does not rely solely on its lipid phosphatase activity. PI(3,4,5)P_3_ was shown to localize also in the nucleus and although its levels were affected by PI3K inhibitors, they remained unaffected after the expression of PTEN implying that nuclear PTEN serves another function in the cell (200). Indeed, localization of PTEN in the nucleus was shown to correlate significantly with the suppressive role of the protein during tumor development independently of its phosphatase activity. To begin with, nuclear PTEN is a protector of genomic stability. Interaction of PTEN with CENP/C promotes chromosomal integrity and positively regulates the expression of Rad51, which reduces the incidence of spontaneous double-strand breaks (DSBs) (191). Additionally, PTEN is required in the nucleus for the formation of the APC/CDH1 complex, which is a regulator of cellular senescence and cell cycle and thus a significant tumor suppressor (193). PTEN also localizes to the nucleolus, and nucleolar PTEN plays an important role in regulating nucleolar homeostasis and maintaining nucleolar morphology (201).

Transport of PTEN in the nucleus was shown to confront to the classic mechanisms and signals for nuclear import. More specifically, the first 32 amino acids of PTEN, which contain a functional PIP2-binding domain that is involved in targeting of PTEN to the plasma membrane, were also shown to contain a functional nuclear localization signal (136, 202). Additionally, import of the protein in the nucleus is dependent on the activity of importins and of Ran-GTPase (136).

Several groups have reported the association of vaults with the nucleus and particularly the nucleoli, the nuclear membrane, and/or the nuclear pore complex and MVPs have been hypothesized as carrier molecules for nuclear–cytoplasmic transport (203). Recently, PTEN was found to associate with MVP (204), whereas more data reveal that this interaction leads to PTEN nuclear import (205). Finally, other researchers suggest that entry of PTEN in the nucleus occurs via diffusion and is not an active process (206). In any case, PTEN nuclear localization is a finely tuned process regulated by ubiquitination (153) as well as by sumoylation of the protein (165).

### Cytoplasmic organelles

An N-terminally extended form of PTEN, which contains 173 additional amino-terminal amino acids (PTEN long; PTEN-L) was found to be expressed and transported in the mitochondria. Translation of PTEN-L is initiated from a CUG codon upstream of AUG and in-frame with the coding region of canonical PTEN. PTEN-L associates with canonical PTEN and the complex serves a role in mitochondrial bioenergetics. Furthermore, depletion of PTEN-L was shown to impair the mitochondrial structure (207).

Apoptosis is a phenotype of cell death and is a process where both mitochondria and endoplasmic reticulum (ER) seem to play a crucial role. A mitochondrial location and accumulation of PTEN after challenging with staurosporine, an apoptosis inducer, were demonstrated after isolation of mitochondrial fractions from primary rat hippocampal cultures. Furthermore, suppression of PTEN expression significantly reduced the increased ROS level detected after the onset of apoptosis and protected hippocampal neurons from STS-induced apoptotic damage (208).

Phosphatase and tensin homolog on chromosome 10 was also shown to localize in the ER and in MAM. PTEN localization at the ER is further increased during calcium (Ca^2+^)-dependent apoptosis induction (209). The regulation of Ca^2+^ concentration is fundamental process for cell metabolism, proliferation, differentiation, and cell death. Elevation in intracellular Ca^2+^ concentration comes either from Ca^2+^ influx from the extracellular space or from Ca^2+^ release from the ER and leads to Ca^2+^ overload in the mitochondria. This induces the swelling of mitochondria, with perturbation or rupture of the outer membrane, and in turn the release of mitochondrial apoptotic factors into the cytosol (209–211). PTEN silencing impairs ER Ca^2+^release, thus reducing the cytosolic and mitochondrial Ca^2+^concentration leading to decreased cellular sensitivity to Ca^2+^-mediated apoptotic stimulation (209). It is proposed that PTEN interacts with and dephosphorylates the inositol 1,4,5-trisphosphate receptors (IP3Rs), which when phosphorylated discharge Ca^2+^from ER. In this concept, ER-localized PTEN regulates Ca^2+^release from the ER in a protein phosphatase-dependent manner (209).

### Extracellular PTEN

Surprisingly, PTEN-L identified in mitochondria was also found to be secreted. More specifically, PTEN-L was found to be localized in exosomes, microvesicles of endosomal origin that are secreted (212). PTEN secretion in exosomes required Nedd4-1 and Nedd4-2 members of the Nedd4 family of E3 ubiquitin ligases as well as Ndfip1, an adaptor protein for the ubiquitin ligases. In addition, lysine 13 within PTEN, which is required for its ubiquitination by Nedd4-1, was required for exosomal transport of PTEN (213). Secreted PTEN enters neighboring cells (212, 213). In fact, the internalization signal for the uptake of PTEN-L into acceptor cells is included in the 173 additional amino acids it contains (212). More importantly, due to the property of PTEN to be secreted, its function is no longer considered to be cell-autonomous. Indeed, uptake of PTEN was shown to antagonize PI3K signaling, to reduce cell proliferation and induce tumor cell death *in vitro* and *in vivo* (130, 214).

## Conclusion

The progress that has been made over the last years in the knowledge of PTEN regulation is remarkable. A wealth of studies has provided novel and unexpected insights into mechanisms of PTEN regulation adding a “positive” complexity in the field. The existence of multiple mechanisms that regulate PTEN expression and activity led to a better understanding of its role in diseases that is expected to contribute to an improvement of the current therapeutic strategies and identification of novel therapeutic approaches. The challenge now is these insights to be placed within a structure to understand how different regulation mechanisms can be targeted by putative new agents in certain diseases.

## Conflict of Interest Statement

The authors declare that the research was conducted in the absence of any commercial or financial relationships that could be construed as a potential conflict of interest.
